# Multi-index comprehensive evaluation model for assessing risk to trainees in an emergency rescue training base for building collapse

**DOI:** 10.1038/s41598-024-55368-z

**Published:** 2024-02-27

**Authors:** Jinyang Li, Zhian Huang, Hongsheng Wang, Hao Ding, Qunlin Jia, Wei Zhao, Tian Le, Danish Jameel, Pengfei Wang

**Affiliations:** 1 State Key Laboratory of High-Efficient Mining and Safety of Metal Mines, University of Science and Technology Beijing, Ministry of Education, Beijing, 100083 China; 2 Institute of Risk Assessment and Control, Guangdong Technology Center of Work Safety, Guangzhou, 510000 China; 3 National Earthquake Emergency Rescue Training Base, Beijing, 100059 China; 4 Work Safety Key Lab on Prevention and Control of Gas and Roof Disasters for Southern Coal Mines, Hunan University of Science and Technology, Xiangtan, 411201 China

**Keywords:** Grey-DEMATEL-ISM model, MICMAC analysis, Emergency training for building collapses, Index system, Factor analysis, Civil engineering, Risk factors

## Abstract

Rescues from building collapse accidents present a significant challenge for China’s emergency rescue system. However, there are also many risk factors in a training scenario, which have been summarized in this study. A hierarchical indicator system for personnel safety was established, including 12 first-level indicators and 23s-level indicators. Then, an improved Grey-DEMATEL-ISM-MICMAC evaluation model was constructed to evaluate the level of risk. Influencing factor scores were determined according to the responses from the questionnaire survey. The influencing degree, influenced degree, centrality, and causality were identified, and the importance, relevance, and clustering of the various factors were obtained after making quantitative calculations. The results showed that the order of priority for solving the essential issues was safety education (A_2_), operating standards and proficiency (A_10_), equipment inspection (A_4_), equipment warehousing maintenance and records (A_21_). The solving of safety education was identified to be the most essential priority. The priority control order of direct causes was Scientific design and construction (A_5_), Potential fixed hazards in the facility (A_12_), Physical fitness of personnel (A_1_), Weather influences (A_18_), and Initiation efficiency of emergency plans (A_20_), and direct control measures for these five factors could achieve a relatively significant effect.

## Introduction

Buildings are necessary for living and working, but they are frequently large and crowded. Their internal structures are becoming increasingly complicated. If a building collapse accident occurs, it is extremely difficult to launch an emergency rescue operation without trained professional assistance. Therefore, collapses are likely to result in mass death and injury^1^. Announced by China’s Ministry of Housing and Urban–Rural Development, there was a total of 245 building collapse accidents of construction industry between 2015 and 2018 in China, resulting in the deaths of 451 people. The average annual death toll was 112.75, and there was an average of 61.25 building collapse accidents per year^2,3^. Furthermore, an average of 1.84 people were killed in each building collapse accident^4^. Frequent building collapse accidents have far-reaching consequences, seriously threatening the lives and property of individuals and bringing about unpredictable direct or indirect economic losses.

In the course of accident rescue operations, the challenging on-site conditions introduce complex risk factors. These factors pose threats to the safety of rescue staff ^5^. Rescue agencies carry out real-life simulation training to learn how to deal with such emergency situations in a timely manner and to reduce the number of casualties and property losses at the rescue site^6^. Presently, the construction of training scenarios for simulating building collapse accidents in China is still in its infancy. In the process of personnel training, various uncertain factors may cause casualties and seriously affect the quality and effectiveness of the training (In this study, ‘personnel’ refers to the person being trained).

In developed countries, previous research on emergency training has already been carried out^7^. Currently, an effective emergency training system is in place, which can cover a vast range of events and offer support in scenarios affecting both commercial enterprises and private individuals^8,9^. Presently, emergency training systems and research into building collapse scenarios in China are relatively immature when compared with researches conducted by European countries and the USA regarding emergency training^10–12^. Multiple training areas can be considered as subdivisions of the emergency training scenarios for use after a building collapse and various training courses are conducted^13^. Training for building collapse search and rescue includes physical fitness, tactical skills, and comprehensive drills. Basic physical training focuses on improving speed, strength, flexibility, and overall fitness to adapt to various needs. Specialized physical training aims at enhancing specific skills like climbing and high-altitude rope rescue. Tactical training targets techniques for personnel search, instrument use, search dogs, safety evaluation, obstacle handling, smoke and toxic rescue environments. Comprehensive drills simulate scenarios with multiple disasters, requiring coordinated operations, reflecting the outcomes of physical and tactical training.

However, the trainees’ safety is affected by many risk factors, such as dust, noise, harmful gases, exposed steel bars, unstable prefabricated panels, flying debris, and other hazards^14^. At the same time, factors such as incomplete emergency plans, a lack of safety awareness, and inadequate safety and supervision measures threaten the trainees’ safety. Moreover, the mutual relationship between these factors is complex^15^. Therefore, it is particularly significant to conduct a safety evaluation of the current system, analyze the interrelationships and substantive effects of the various influencing factors in depth, and provide effective safety guarantees for personnel. Scholars have conducted seminal research into the causes of building collapse and the need for emergency rescue^16^, but they have not yet created a comprehensive multi-index safety evaluation procedure for the emergency training system related to building collapse scenarios. Therefore, there is a lack of identification and effective control of factors affecting the trainees’ safety. Furthermore, the indicator system and evaluation model are also incomplete.

In this study, the building collapse emergency training base was taken as the evaluation object. Through field investigation and interviews, risk identification was carried out from the perspective of “Human–Machine–Environment–Management”. In addition, the safety guarantee index system for a building collapse emergency training base is established and a novel multi-index comprehensive evaluation model (Grey-DEMATEL-ISM-MICMAC evaluation system) was established. The logical relationships between the various safety factors and the importance of those factors that affect trainees’ safety during emergency rescue training were thoroughly analyzed. Then, quantitative calculation and verification of the base itself were carried out. Based on the importance, relevance, and clustering of the identified factors, the order of priority control of these factors was determined, and the corresponding countermeasures and suggestions are proposed in this study. The research results can provide a reference for guaranteeing personnel safety in an emergency training base for building collapse events and will improve the personnel safety guarantee of the emergency training system.

## Methodology

Different theoretical methods can be employed for comprehensive evaluation. The Chinese scholar, Deng Julong^17^, proposed the grey system theory, while the fuzzy set theory was propounded by Professor L.A. Zadeh^18^ in 1965, followed by Saaty^19^, who put forward the system engineering theory in 1977.

Safety in the building collapse emergency rescue training base is affected by multiple factors, and there is a certain degree of dependence and feedback interaction that occurs between these factors^20^. However, the traditional linear analysis model has some defects, which causes only partial analysis of this complex system^21,22^. Considering the safety index number of the training base and the uncertainty of the index regarding comprehensive evaluation results, the grey system theory, DEMATEL (the decision-making trial and evaluation laboratory), ISM (interpretative structural modeling), and MICMAC (impact matrix cross-reference multiplication, applied to a classification) were used to comprehensively evaluate the risks inherent in an emergency rescue training base for dealing with building collapses.

### Grey system theory

Grey system theory is a further development of system theory. It provides an analytical and modeling method for dealing with fuzziness in decision problems that handle discrete data and incomplete information^23^. A modified method of converting fuzzy data into crisp scores (CFCS) is used in defuzzification, which is adopted in the grey aggregation process of this study. Many scholars agree that this method is more effective for obtaining clear values, compared to other methods^24,25^.

Due to the uncertainty and fuzziness inherent in expert evaluation, this study defines $\otimes x_{ij}^{p}$ as the grey number of evaluators (decision-makers), *p*. This represents the evaluation score of decision-makers *p (p* = *1, 2…, n)* regarding the degree to which factor* i* affects factor* j*, as calculated using Eq. (1):

1 $$ \otimes x_{ij}^{p} = [\underline{ \otimes } x_{ij}^{p} ,\overline{ \otimes } x_{ij}^{p} ] $$

where *p* represents the number of evaluators (decision-makers), *p* = *1, 2, 3… m*; $\underline{ \otimes } x_{ij}^{p}$ is the minimum grey value of the degree to which safety guarantee factor* i* affects factor* j*; $\overline{ \otimes } x_{ij}^{p}$ is the maximum grey value of the degree to which the safety guarantee factor* i* affects factor* j*.

The modified CFCS defuzzification method consists of three main steps, as shown below.

*Step 1:* Normalization:

2 $$ \underline{ \otimes } \widetilde{x}_{ij}^{p} = (\underline{ \otimes } x_{ij}^{p} - \mathop {\min }\limits_{j} \otimes x_{ij}^{p} )/\Delta_{\min }^{\max } $$

3 $$ \overline{ \otimes } \widetilde{x}_{ij}^{p} = (\overline{ \otimes } x_{ij}^{p} - \mathop {\min }\limits_{j} \otimes x_{ij}^{p} )/\Delta_{\min }^{\max } $$

4 $$ \Delta_{\min }^{\max } = \mathop {\max }\limits_{j} \overline{ \otimes } x_{ij}^{p} - \mathop {\min }\limits_{j} \underline{ \otimes } x_{ij}^{p} $$

where $\overline{ \otimes } \widetilde{x}_{ij}^{p}$ is the normalized maximum grey number; $\underline{ \otimes } \widetilde{x}_{ij}^{p}$ is the normalized minimum grey number.

*Step 2:* Computing the total normalized crisp value:

5 $$ Y_{ij}^{p} = \frac{{\underline{ \otimes } \widetilde{x}_{ij}^{p} (1 - \underline{ \otimes } \widetilde{x}_{ij}^{p} ) + (\overline{ \otimes } \widetilde{x}_{ij}^{p} \times \overline{ \otimes } \widetilde{x}_{ij}^{p} )}}{{1 - \underline{ \otimes } \widetilde{x}_{ij}^{p} + \overline{ \otimes } \widetilde{x}_{ij}^{p} }} $$

*Step 3:* Computing the crisp values:

6 $$ Z_{ij}^{p} = \mathop {\min }\limits_{j} \underline{ \otimes } x_{ij}^{p} + Y_{ij}^{p} \times \Delta_{\min }^{\max } $$

### Decision-making trial and evaluation laboratory method (DEMATEL)

Many scholars have adopted the DEMATEL method to carry out research and accurately identify the correlation (causality) between various factors in the system^26^. Wang et al.^27^ combined the important analysis of satisfaction with the DEMATEL method and proposed a new model, which was then applied to an advanced facility design project. DEMATEL belongs to the structural modeling method (See supplementary material for definitions). It utilizes the knowledge and experience of experts and applying directed graph theory to clearly reflect the interdependent relationships. Then it influences values among various complex factors within a system: (1) problem analysis and factor set establishment; (2) modeling of direct impact relationships; (3) normalization of impact relationships; (4) modeling of total relation matrix; (5) calculation of impact degrees.

### Interpretative structural modeling (ISM)

**Figure 1:**
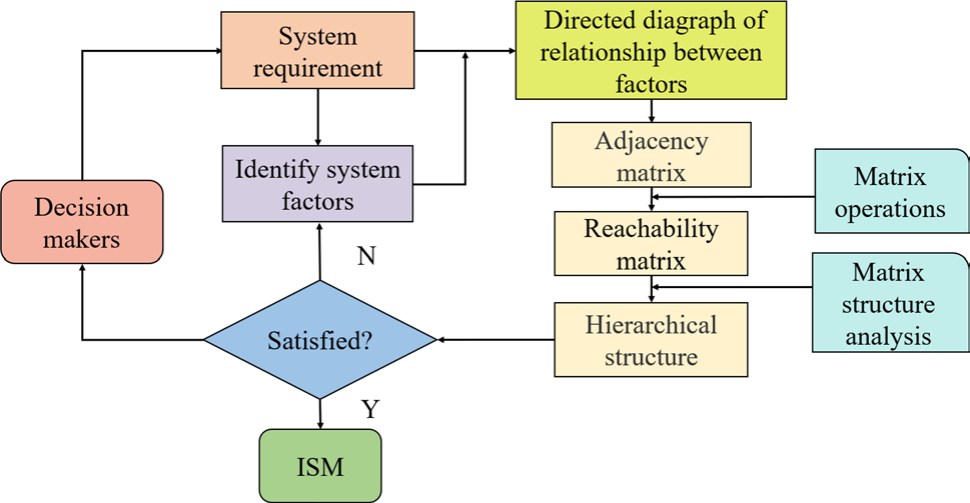
The ISM model.

The ISM system was proposed by Professor J. Warfield in 1973. It belongs to a type of structured technical model that can analyze problems at different macro and micro levels^28,29^. However, at this time, ISM is rarely used in the evaluation of personnel safety and protection in the context of emergency rescue training. The steps for ISM are shown in Fig. 1. The modeling steps of ISM are as follows:

(1) Set up the ISM implementation evaluation team; (2) Analyze system requirements and select influencing factors that lead to key problems; (3) Enumerate correlations among various risk factors that affect each other; (4) The adjacency matrix and reachability matrix are established according to the correlation among various factors; (5) Construct the structural model, after decomposing the reachable matrix; (6) An explanatory structural model is established according to the structural model and the conclusion is analyzed.

### Cross-impact matrix multiplication applied to classification (MICMAC)

The MICMAC method calculates the influence degree of each factor in the reachability matrix. The main purpose behind the MICMAC method is to analyze the influence and dependence of factors in a complex system by classifying the driving power and dependence in the system. The main purpose of MICMAC is to analyze the influence and dependence between factors in complex systems by classifying the driving forces and dependencies of factors within the system. Driving force represents the factor’s ability to influence other factors and is also an accessible element in ISM. Dependence represents the degree to which the factor is affected by other factors. It is the antecedent element in ISM.

### Multi-index comprehensive evaluation model based on grey-DEMATEL-ISM-MICMAC theory

**Figure 2:**
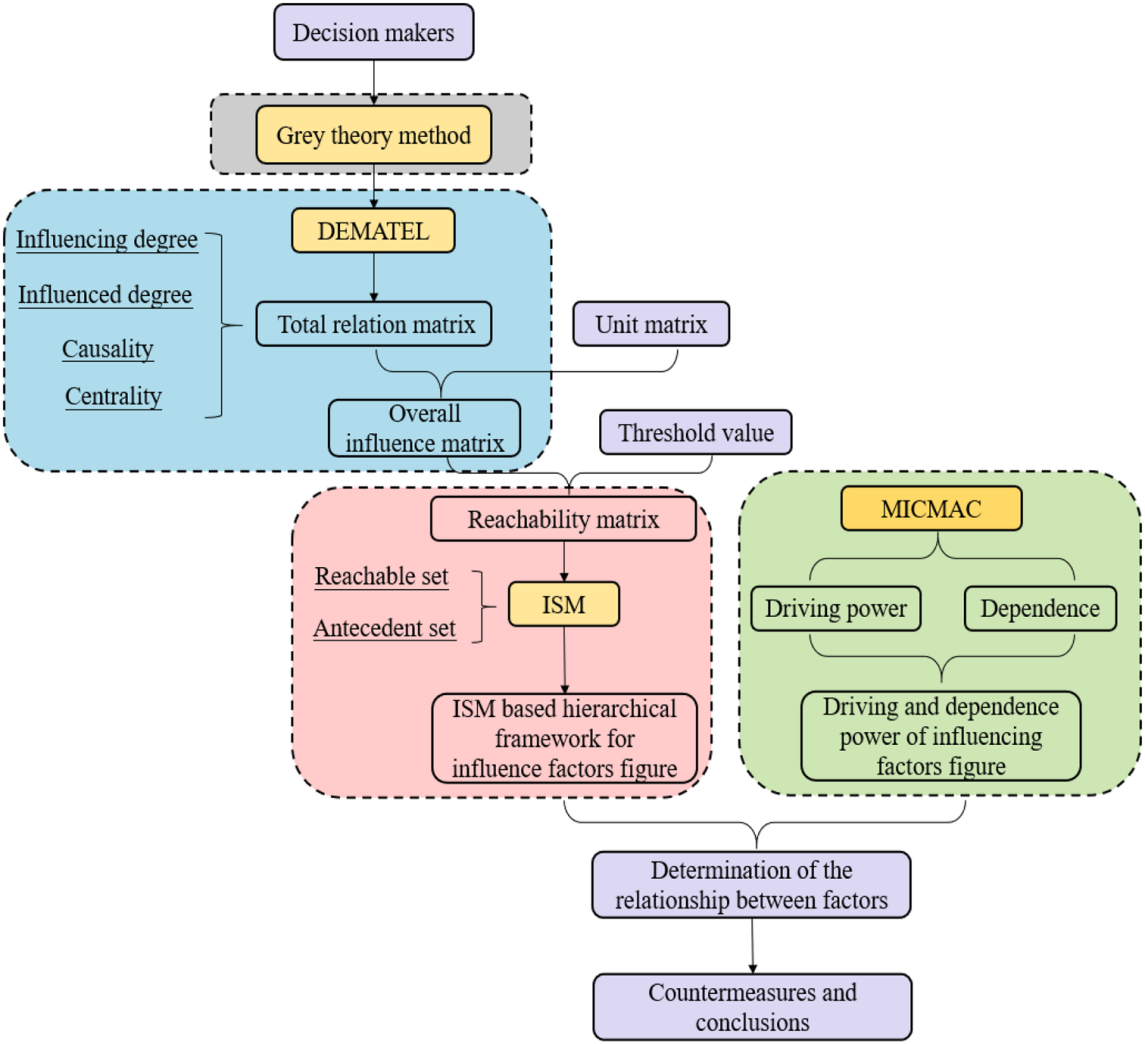
Modified grey-DEMATEL-ISM-MICMAC model flowchart.

Grey-DEMATEL-ISM-MICMAC evaluation model for the factors influencing trainee’s safety guarantees in emergency rescue training is shown in Fig. 2.

Grey system theory considers the concept of a fuzzy condition. Its main advantage is fuzzy set theory^30^. The DEMATEL method is insufficient to deal with uncertainties, nor can it express the fuzzy value around a given discrete value^31^. Therefore, both grey theory and DEMATEL are integrated into this study. Although ISM is able to divide the system hierarchy, it cannot clarify the degree of influence of the factors in the system^32,33^. Based on the hierarchical structure of factors determined by the Grey-DEMATEL-ISM method, this study further analyzes the position and action of factors in the system by combining the method with MICMAC. The driving power and dependence factors are calculated and then the factors are classified, in order to understand the essential role of each factor.

## Multi-index comprehensive evaluation model for an emergency rescue training base for building collapse scenarios

The process of applying the multi-index comprehensive evaluation model for emergency rescue training in building collapse scenarios is as follows.

### Selection of evaluation objects

**Figure 3:**
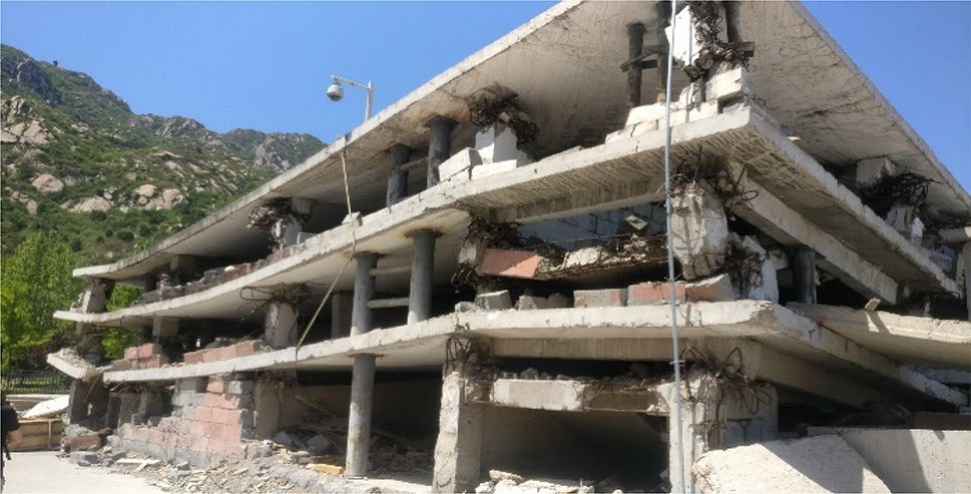
Rescue training in a ruined building.

**Figure 4:**
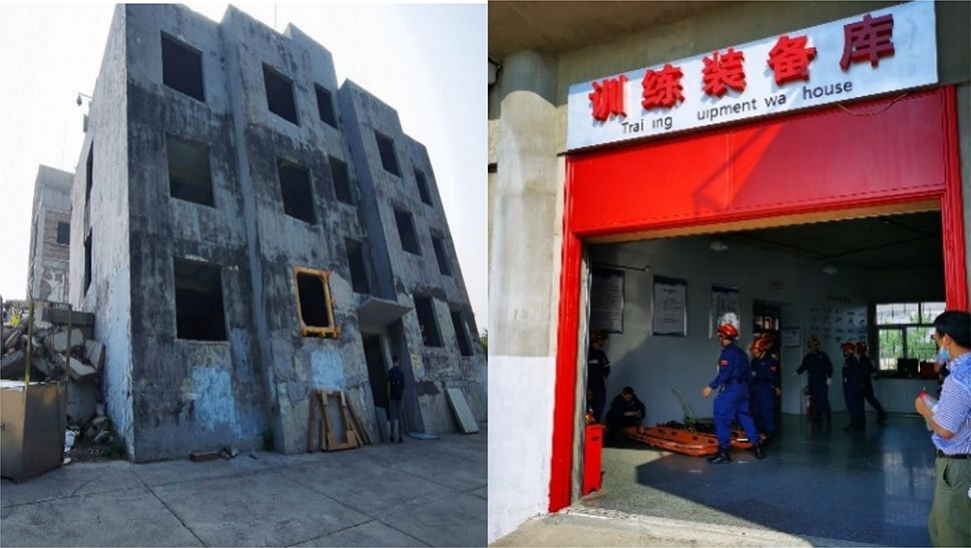
The inclined building and training equipment warehouse.

For this study, an emergency rescue training base was selected as the subject. The base incorporates a teaching complex, a virtual simulation training hall, a ruined structure for earthquake rescue training ruins (see Fig. 3), a rescue training equipment warehouse, and an inclined building (see Fig. 4), among other elements.

### Index system for the influencing factors in personnel safety guarantees

#### Identification of risk factors in the training system

The causes of building collapse are intricate, primarily influenced by factors such as earthquakes, fires, explosions, construction issues, or engineering quality problems. The emergency training for building collapse scenarios differs from actual building collapse sites. The training is mainly on handling large collapsed components, training in confined underground spaces, self-protection and safety awareness training.

Based on field interviews, investigations, and expert opinions, this study analyzes the risks and harmful factors based on accident cause theory and the “Human–Machine–Environment–Management” theory. The main factors threatening the trainees’ safety are analyzed from the perspective of four aspects: (1) the unsafe behavior of trainees, (2) the unsafe state of machinery, (3) management defects (see Supplementary material for definitions), and (4) the harmful environment.

#### Index system of the influencing factors

The safety evaluation index system for the emergency rescue training base for building collapse scenarios is shown in Table 1. A detailed explanation of the indicators is shown in Supplementary material. The aim of this model is to evaluate factors that may cause harm to trainees during the training process in the building collapse training base. The model incorporates 12 first-level indexes, which are divided into 23s-level indexes.

**Table 1:** Safety evaluation index system of the emergency training base for building collapse scenarios.

Influencing factors on the emergency training base for building collapse	First-level indexes	Second-level indexes
	Qualifications of relevant personnel (M_1_)	Physical fitness of personnel (A_1_)
		Safety education (A_2_)
	Equipment (M_2_)	Equipment function (A_3_)
		Equipment inspection (A_4_)
	Training facilities stability (M_3_)	Scientific design and construction (A_5_)
		Facility inspection and maintenance (A_6_)
	Personnel and organizational structure (M_4_)	Personnel ratio (A_7_)
		Organization system (A_8_)
	Health care system (M_5_)	Emergency supplies and plans (A_9_)
	Personnel fault (M_6_)	Operating standards and proficiency (A_10_)
		Safety supervisor competence (A_11_)
	Facility risk (M_7_)	Potential fixed hazards in the facility (A_12_)
		Detection and monitoring coverage (A_13_)
	Equipment reliability (M_8_)	Equipment protection effectiveness (A_14_)
		Communication anti-interference ability (A_15_)
		Intact personal protective equipment (A_16_)
	Operating environment risk (M_9_)	Chronic occupational hazards (A_17_)
		Weather influences (A_18_)
	Implementation of a safety management system (M_10_)	On-site management and control (A_19_)
		Initiation efficiency of emergency plans (A_20_)
	Maintenance, recovery, and recording (M_11_)	Equipment warehousing maintenance and records (A_21_)
		Recovery and recording after training (A_22_)
	Mental health (M_12_)	Rotation time and mental burden (A_23_)

The evaluation index system for assessing the safety of personnel in the emergency training base for building collapse scenarios is not rigid; it changes with time, space, and scenario characteristics. Therefore, necessary and appropriate adjustments should be made in specific areas to adapt to real-world situations. Specifically, these adjustments may include adaptive changes to factors such as different geographical regions, climate conditions, types of building structures, and rescue equipment configurations. In addition, as technology progresses and rescue equipment is updated, the evaluation index system also needs to be continuously revised and improved. For example, new life detectors and other equipment may have an impact on the evaluation of personnel safety. Therefore, the system should update the evaluation index system to ensure that it always reflects current best practices.

### Establish the decision group

To determine the most suitable factors for this study, a team of four members was assembled. This team consisted of three academic experts with over five years of research experience in emergency training-related fields, and a specialist in field emergency rescue training with more than 10 years of practical training experience.

A detailed questionnaire was designed for the four experts regarding the influencing relationship between emergency training and safety guarantee factors. In the questionnaire, a 5-level scale was used to estimate the direct influencing relationship between the different factors. An evaluation score matrix of n × n was obtained.

### Grey-DEMATEL-ISM-MICMAC methodology

Using the modified Grey-DEMATEL-ISM-MICMAC methodology, our study has identified influencing relationships among the factors affecting an emergency rescue training base for building collapse scenarios. Grey-DEMATEL-ISM-MICMAC system includes two main aspects: (1) Grey-DEMATEL-ISM methodology is used to establish a hierarchical structure model for each factor in the system, (2) MICMAC is used to cluster each factor, and (3) the role of each factor in the system is determined via comprehensive analysis.

The steps involved in the developed methodology are discussed below. Among them, steps 1 to 3 are gray method of processing data. Steps 4 to 7 belong to DEMATEL method. Step 8 is ISM model and step 9 is MICMAC method.

*Step 1:* Define the safety factor set.

The factors affecting trainee’s safety in the emergency training system regarding building collapses are defined as *A* = {*A*_*1*_*, A*_*2*_*, …, A*_*23*_}. In this study, a 5-level scale is adopted. The direct influence degree of factor *I* on factor *j* is given a score from 0 to 4. The experts’ evaluation of the linguistic terms and grey numbers is shown in Table 2. Since the score value of each factor is uncertain, the interval grey number processing technique is adopted to improve the objectivity of the data results^33^.

**Table 2:** Experts’ evaluation of the linguistic terms and grey numbers.

Linguistic terms	Evaluation score	Grey numbers
No influence (N)	0	[0, 0]
Very low influence (VL)	1	[0, 0.25]
Low influence (L)	2	[0.25, 0.5]
High influence (H)	3	[0.5, 0.75]
Very high influence (VH)	4	[0.75, 1]

*Step 2:* Determine the influence relationship matrix between the factors.

The evaluation scores given by each expert were transformed into grey numbers: $\otimes x_{ij}^{p} = [\underline{ \otimes } x_{ij}^{p} ,\overline{ \otimes } x_{ij}^{p} ]$. After calculating the crisp grey numbers using Eqs. (1)–(6), the initial influence relational matrix, Z^p^, was obtained for each expert on the factor set. In the same way, expert 2, expert 3, and expert 4 have been assigned the initial influence relationship matrixes, Z^2^, Z^3^, and Z^4^, respectively.

*Step 3:* Calculate the weighted influence relationship matrix, Z.

Since the experts selected for this study possess almost the same knowledge levels in terms of experience, cognition, and research depth in the field of emergency training for building collapse scenarios, it is equally important to set a value for the influence of expert opinions and assign the same weight after comprehensive consideration: $w_{i} = 0.25(i = 1,2,3,4)$.

*Step 4:* Calculate the normalized influence relationship matrix, C.

Using Eqs. (7) and (8), the normalization influence relationship matrix, C, is calculated as follows:

7 $$ S = \frac{1}{{\mathop {\max }\limits_{1 \le i \le n} \sum\nolimits_{j = 1}^{n} {\overline{z}_{ij} } }},i,j = 1,2,...,n $$

8 $$ C = S \cdot Z. $$

After calculation, it was determined that *S* = 0.1835.

*Step 5:* Obtain a comprehensive influence relationship matrix, T.

The comprehensive influence relationship matrix T ($T = [t_{ij} ]_{23 \times 23}$) is obtained via Eq. (9):

9 $$ T = \sum\nolimits_{K = 1}^{\infty } {C^{K} } \to T = C(I - C)^{ - 1} $$

where *i* represents the unit matrix.

Then, influencing degree (*D*_*i*_), influenced degree (*E*_*i*_), centrality (*F*_*i*_), and causality (*M*_*i*_) are calculated, as below:

10 $$ D_{i} = \sum\limits_{i = 1}^{n} {t_{ij} (i = 1,2, \ldots ,n)} $$

11 $$ E_{i} = \sum\limits_{j = 1}^{n} {t_{ji} (i = 1,2, \ldots ,n)} $$

12 $$ F_{i} = D_{i} + E_{i} ,(i = 1,2, \ldots,n) $$

13 $$ M_{i} = D_{i} - E_{i} ,\quad (i = 1,2, \ldots ,n). $$

The influence degree (*D*_*i*_) is the comprehensive influence value of factor *i* on other factors. It is given by the sum of the value of the elements in each row of matrix T. The influenced degree (*E*_*i*_) represents the comprehensive influence value of other factors on factor *i* and is given by the sum of the element values of each column of matrix T. The centrality (*F*_*i*_) indicates the importance of factor* i* to the entire system. The greater the centrality, the more obvious the influence of this factor in the system; this factor can be considered the main factor. If causality (*M*_*i*_) > 0, this means that factor* i* has a great influence on the other factors and can be identified as a causal factor; conversely, if causality (*M*_*i*_) < 0, this means that other factors easily affect factor *i*. It can be identified as a result factor.

Influencing degree (*D*_*i*_), influenced degree (*E*_*i*_), centrality (*F*_*i*_) and causality (*M*_*i*_) of factors are shown in Table S1 in the Supplementary Material.

*Step 6:* Calculate the overall influence relationship matrix, H.

The overall influence relationship matrix H ($H = [h_{ij} ]_{23 \times 23}$) is calculated using Eq. (14). The comprehensive influence matrix T reflects the relationships between and the degrees of mutual influence among the various factors. However, this does not take into account the effect of a factor on itself. The overall influence relationship matrix H reflects the overall influence relationship among all factors in the system, which not only reflects the mutual influences of factors but also reflects the influence relationship of factors with themselves.

14 $$ H = T + I $$

*Step 7:* Determine the reachability matrix, K.

The reachability matrix, K ($K = [k_{ij} ]_{23 \times 23}$), is calculated using Eq. (15):

15 $$ k_{ij} = \left\{ \begin{array}{ll} 1,\quad h_{ij} \ge \lambda \hfill \\ 0,\quad h_{ij} \le \lambda \hfill \\ \end{array} \right.(i,j = 1,2, \ldots ,n). $$

From the overall influence matrix, H, the reachability matrix, K, is calculated with a given threshold value of λ, where $k_{ij} = 1$. This means that factor A_i_ can influence factor A_j_. $k_{ij} = 0$ means that factor A_i_ cannot influence factor A_j_.

Choosing a reasonable threshold value, λ, is the key to transforming the overall influence matrix H into a reachability matrix, K. Setting the threshold value, λ, eliminates less influential relationships and simplifies the complex system structure, which is conducive to an effective division of the system hierarchy. Based on numerous test results, expert advice, and practical requirements, λ was set at 0.0098 in this condition.

*Step 8:* Construct an ISM model of various factors in the system.

According to the reachable matrix K, the ISM model is drawn up by hierarchical division. First, the reachable set, *R*_*i*_, and the antecedent set, *W*_*i*_, of the factors are determined. The reachable set represents all factors directly or indirectly influenced by factor *A*_*i*_, while the antecedent set represents all the factors directly or indirectly influencing factor *A*_*i*_. The calculation of the reachable set and the antecedent set is achieved as shown in Eqs. (16) and (17):

16 $$ R_{i} = \left\{ {\left. {A_{j} } \right|A_{j} \in A,k_{ij} = 1} \right\},(i,j = 1,2, \cdot \cdot \cdot ,n) $$

17 $$ W_{i} = \left\{ {\left. {A_{j} } \right|A_{j} \in A,k_{ji} = 1} \right\},\quad (i,j = 1,2, \ldots ,n). $$

After obtaining the reachable set, *R*_*i*_, and the antecedent set, *W*_*i*_, Eq. (18) is used to verify factor *A*_*i*_ in the system. If Eq. (18) holds true, then the reachable set of factor *i* is included in the antecedent set of the factor, as shown in Table S2 in the Supplementary Material. According to the ISM structural model, the hierarchy of the factors affecting the guarantee of personnel safety in emergency rescue training can be determined.

18 $$ R_{i} = R_{i} \cap W_{i} ,i = 1,2, \ldots ,n $$

As shown in Table S2 in the Supplementary Material, the factor sets that satisfy $R_{i} = R_{i} \cap W_{i} = (A_{1} ,A_{5} ,A_{12} ,A_{18} ,A_{20} )$ form the underlying elements, and these factor sets form the direct cause layer (the underlying layer). Together, these factors constitute the first layer, L_1_, of the multi-level hierarchical structure model, namely, $L_{1} = (A_{1} ,A_{5} ,A_{12} ,A_{18} ,A_{20} )$.

**Figure 5:**
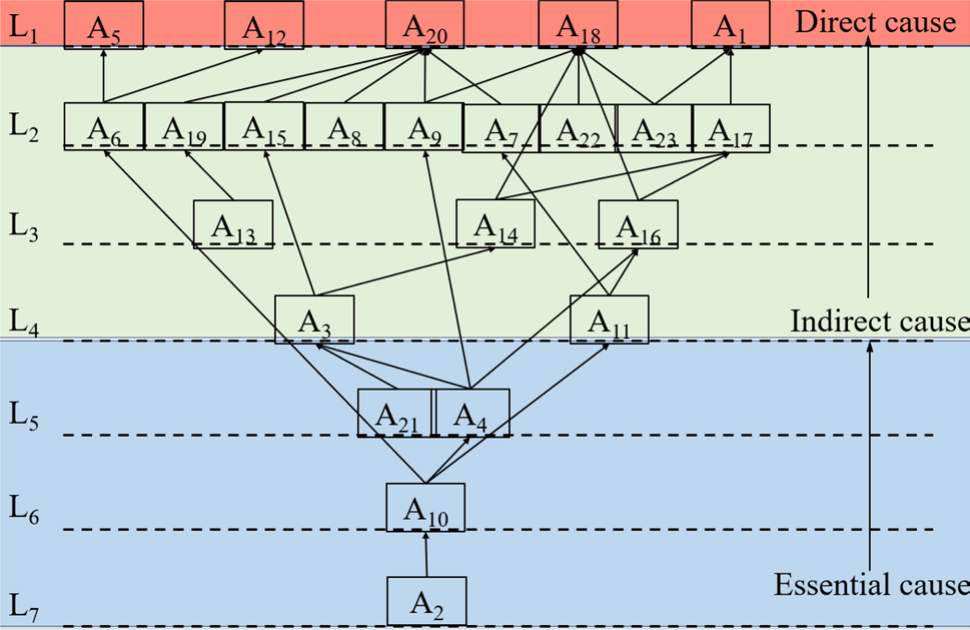
A hierarchical model of the influencing factors of emergency rescue training in building collapse scenarios.

Then, the corresponding row and column are deleted from the matrix, K. According to the division of the above set, verification continues on the outcome of Eq. (18). Based on this verification, the factors contained in L_2_, L_3_, and L_S_ are successively determined until all rows in the table are marked out. Finally, the ISM model of each influencing factor in the system can be drawn up according to hierarchical division and the reachable matrix, K. As shown in Fig. 5, this model can clearly reflect the various factors influencing personnel safety guarantees in emergency rescue training and the levels of their mutual influence mechanism.

*Step 9:* Combine MICMAC to divide the factors into clusters and generate a driving power and dependence power for the influencing factors. Driving force and dependence divide all factors into four clusters: autonomous factors, dependent factors, linkage factors, and independent factors.

(1) Autonomous factors.

The driving force and dependence of such factors are low. They are close to the origin of the coordinate axis. It means that they have poor correlation with other factors. They are not easily affected by other factors and are not easy to affect other factors.

(2) Dependent factors.

The driving force of the factors is weak, but they have strong dependence. It means that they have a small impact on other factors, but they are easily affected by other factors. To a large extent, the factors will be affected by changes in other factors.

(3) Linkage factors.

The driving force and dependence of the factors are large. It means that they are usually unstable. Any effect on the factors will have an impact on other factors. Because of the strong dependence, it is easy to have consequences on itself. Linkage factors can be considered unstable factors.

(4) Independent factors.

They have strong driving force but weak dependence. It means that they can easily affect other factors in the system. But the influence from other factors is little. This type of factor is the basic condition to the influence of other factors. It is difficult to control it indirectly through other factors. Therefore, they are also called key factors.

According to the reachable matrix, K, obtained in the previous step, the driving power (*DR*_*i*_) and dependence (*DE*_*i*_) of each factor of the system are calculated, as shown in Eqs. (19) and (20):

19 $$ DR_{i} = \sum\limits_{j = 1}^{n} {k_{ij} ,i = 1,2,\ldots } ,n $$

20 $$ DE_{i} = \sum\limits_{i = 1}^{n} {k_{ij} ,j = 1,2,\ldots } ,n $$

where *DR*_*i*_ represents the driving degree of this factor regarding other factors in the system; *DE*_*i*_ indicates how dependent the factor is on other factors in the system.

**Figure 6:**
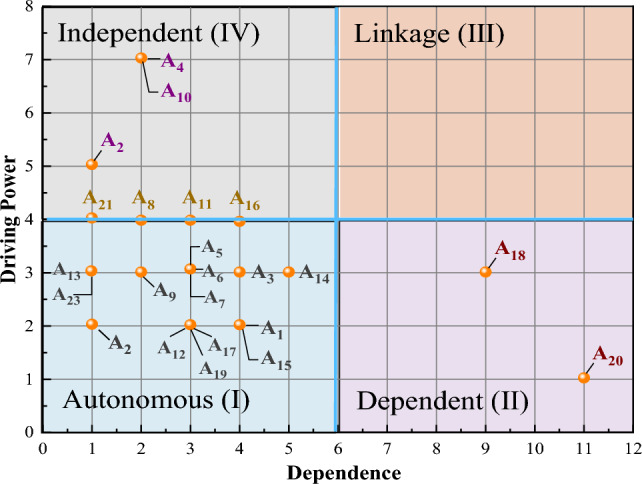
Driving power and dependence of the influencing factors.

Taking the dependence as the abscissa and the driving power as the ordinate, a chart with the driving power and dependence power of influencing factors in the system is drawn up. According to the driving power and dependence of each factor, all factors in the system are divided into four categories: autonomous factors (I), dependent factors (II), linkage factors (III), and independent factors (IV), as shown in Fig. 6.

## Analysis of the findings

### Analysis of grey-DEMATEL model

As can be seen from Table S2 in the Supplementary Material, the factors with the largest influence degrees are: A_4_, A_10_, A_2_, A_8_, A_11_, A_9_. These are the main factors that lead to a guarantee of the safety of personnel in the emergency rescue training base who are experiencing a building collapse scenario.

In terms of the influenced factors (*E*_*i*_), A_20_, A_18_, A_14_, A_16_, and A_17_ rank highly. Promoting these factors may enhance the standard of safety in the emergency rescue training base.

In terms of centrality (*F*_*i*_), the initiation efficiency of emergency plans (A_20_) plays the most significant role in the system, followed by weather influences (A_18_), operating standards and proficiency (A_10_), equipment inspection (A_4_), equipment protection effectiveness (A_14_), and intact personal protective equipment (A_16_).

Causality (*M*_*i*_) shows the position of certain factors in the system, indicating their effect on the system. When *M*_*j*_ > 0, these are known as cause factors. Equipment inspection (A_4_), safety education (A_2_), operating standards and proficiency (A_10_), equipment warehousing maintenance and records (A_21_), and emergency supplies and plans (A_9_) have high centrality. Consequently, they significantly affect other factors. This analysis shows that these six factors are more active than the remaining factors and should be given more attention in the training process. When *M*_*j*_ < 0, these are known as effect factors. Among these, the factors on initiation efficiency of emergency plans (A_20_), weather influences (A_18_), chronic occupational hazards (A_17_), and equipment protection effectiveness (A_14_), are influenced strongly. Compared with other factors, they have strong volatility and poor stability. In particular, the factor of initiation efficiency of emergency plans (A_20_) is the most vulnerable to the influence of other factors and is the most difficult factor to control in terms of management.

### Analysis of the ISM

As shown in Fig. 5, the modified ISM divides the 23 factors into 7 levels, and there is a causal relationship between these levels.

#### Direct causes

The factors on layer L_1_ directly influence the safety of emergency training. Moreover, these factors may threaten the lives and health of trainees. They include the physical fitness of personnel (A_1_), scientific design and construction (A_5_), potential fixed hazards in the facility (A_12_), weather influences (A_18_), and the initiation efficiency of emergency plans (A_20_). These factors have the potential to be a direct cause of accidents in emergency training. When they are triggered, these factors have a strong impact on training safety, directly leading to accidents.

#### Indirect causes

Layers L_2_, L_3_, and L_4_ in the improved ISM model form the intermediate layers. The influencing factors in the intermediate layer are relatively complex. They are indirect factors that lead to accidents. Moreover, although difficult to address, they are key points in ensuring the normal development of emergency training. Among them, A_8_, A_13_, A_22_, and A_23_ have no direct influence on other factors; these are obstacle factors that must be solved independently.

#### Essential causes

The essential causes (L_5_, L_6_, and L_7_) are as follows: safety education (A_2_), equipment inspection (A_4_), operating standards and proficiency (A_10_), and equipment warehousing maintenance and records (A_21_). These four factors affect the other factors of each layer through transitivity. In particular, safety education (A_2_) appears at the bottom of the model. It is the primary factor that requires the greatest attention in terms of personnel safety and it is a profoundly influencing factor.

#### Analysis of the MICMAC model

In terms of emergency training, the influencing factor cluster can be divided into autonomous factors, dependent-factor-linkage factors, and independent factors. Obviously, those factors related to autonomy are the largest, followed by the dependent factors and independent factors. There is no linkage factor.

#### Autonomous factors (I)

In total, there are 14 factors (A_1_, A_3_, A_5_, A_6_, A_7_, A_9_, A_12_, A_13_, A_14_, A_15_, A_17_, A_19_, A_22_, and A_23_) that are part of the autonomous factor group. The driving power and dependence in this cluster are relatively small, indicating that they are not easily influenced by other factors and do not easily influence other factors. In the ISM structure model, these factors are mostly located in the direct causes layer and in the upper part of the indirect causes layer. Therefore, autonomous factors should be the first that are addressed to govern emergency training safety.

#### Dependent factors (II)

There are two factors (A_18_ and A_20_) that belong to the dependent factor group, which have a high dependence power and a low driving power. These factors need to be controlled by the other factors. Solutions regarding these factors will depend on the solutions of other factors. At the same time, these factors are located in the first layer of the ISM model, that of direct causes. In addition, the influenced degree of the two factors is the high, which is also consistent with this result.

#### Linkage factors (III)

Factors classified as linkage factors have both stronger dependence power and driving power. In our study, none of the factors were classified as linkage factors.

#### Independent factors (IV)

There are three factors (A_2_, A_4_, and A_10_) belonging to the independent factor group, which have high driving power and low dependence power. These are all at the bottom of the ISM model (L_5_, L_6_, and L_7_) and are the essential causes affecting emergency training safety. Furthermore, these three factors have a great influence on other factors. They are the basic conditions that influence other factors.

In addition, A_8_, A_11_, A_16_, and A_21_ are located at the junction of the autonomous factor (I) and independent factor (IV). It means that they have both autonomous and independent factor characteristics. The driving power of these four factors is equal. And the order of dependence power is A_16_, A_11_, A_8_, and A_21_. Among them, A_16_, A_11_, and A_8_ are grouped with the indirect causes, while A_2_ is an essential cause. A_21_ and A_8_ are relatively independent factors. Therefore, the priority of these four factors is as follows: A_21_ > A_8_ > A_11_ > A_16_.

### Countermeasures for emergency rescue training

For the four essential causes, the priority is A_2_ > A_10_ > A_4_ > A_21_. Safety education (A_2_) is the primary factor that should receive attention during emergency training in a building collapse scenario. With the need for advanced building collapse emergency training courses and suitable training courses in China should be developed. Safety manuals, classroom teaching, VR virtual training, safety awareness, and the level of professional knowledge should be improved. Operating standards and proficiency (A_10_) are directly influenced by safety education (A_2_). Proficiency needs to be improved through repeated training conducted by the trainer. Improving this safety factor can effectively improve other factors on the intermediate layers. In terms of the inspection of equipment before training, recruiting personnel with a solid theoretical knowledge foundation and richness of practical experience to carry out the functional and performance testing of equipment is vital. However, equipment warehousing maintenance and records (A_21_) are not affected by these deep factors. Therefore, as an independent factor, priority should be given to safety and control measures.

According to the 14 factors of indirect causes, factors that need to be addressed (in priority order) are detection and monitoring coverage (A_13_), the organization system (A_8_), recovery and recording after training (A_22_), rotation time, and the mental burden (A_23_). These four indirect factors are not influenced by the deep factors. They have a direct or indirect influence on the L_2_ and L_1_ layer factors. They are relatively independent and belong to the autonomous factor group, which should be given greater research attention.

As for the five direct cause factors of the L_1_ layer, the priority order is scientific design and construction (A_5_), potential fixed hazards in the facility (A_12_), the physical fitness of personnel (A_1_), weather influences (A_18_), and the initiation efficiency of emergency plans (A_20_). These five factors are all affected by the intermediate layer or the bottom layer and will then directly influence emergency training safety.

## Conclusions

The safety of an emergency rescue training base for building collapse scenarios is important during the training process for trainees. In this study, an improved Grey-DEMATEL-ISM-MICMAC model was constructed to evaluate the building collapse scenarios. The influencing mechanism between the different factors involved was studied. It provides an example of the safety evaluation process in emergency training bases.

1. Through Grey-DEMATEL model, the influence relationship and influence degree among the various factors were revealed. Combined with ISM, the hierarchical structure was divided up to determine the correlations between factors. Factors were classified into essential causes, indirect causes, and direct causes. Finally, the driving force and dependence of each factor were obtained using MICMAC.
2. An emergency rescue training base in China was taken as an example. A safety evaluation index system of the training base for emergency rescue after a building collapse was established. The indexes and scoring system were determined by expert scoring, and the importance, relevance, and clustering of all factors were obtained after quantitative calculation.
3. Countermeasures for emergency rescue training were proposed for essential causes, direct causes, and indirect causes. Among the essential causes, the priority factors for solving the safety issues are A_2_, A_10_, A_4_, and A_21_. In particular, A_2_ is the primary factor that requires the greatest attention. The priority order of indirect causes is A_13_, A_8_, A_22_, and A_23_. The priority order of direct cause and factor control is A_5_, A_12_, A_1_, A_18_, and A_20_. Taking direct control of these five factors can achieve obvious safety improvements.

### Supplementary Information

Supplementary Information.

## Data Availability

The datasets used and/or analysed during the current study available from the corresponding author on reasonable request.
