# Supplementary material for: Multi-index comprehensive evaluation model for assessing risk to trainees in an emergency rescue training base for building collapse
Source: Sci Rep. 2024 Feb 27;14:4792. doi: 10.1038/s41598-024-55368-z (PMC10899225; doi:10.1038/s41598-024-55368-z)
Supplement: Supplementary file 1 — Supplementary Information. [file 41598_2024_55368_MOESM1_ESM.docx]

**Multi-Index Comprehensive Evaluation Model for Assessing Risk in an Emergency Rescue Training Base for Building Collapse**

Jinyang Li^a^ , Zhian Huang^a^, Hongsheng Wang ^a,b,*^, Hao Ding^a,b,**^, Qunlin Jia^c^, Wei Zhao^a^, Tian Le^a^_,_ Danish Jameel^a^, Pengfei Wang^d^

^a^*State Key Laboratory of High-Efficient Mining and Safety of Metal Mines, University of Science and Technology Beijing, Ministry of Education, Beijing 100083, China*

^b^ *Institute of Risk Assessment and Control, Guangdong Technology Center of Work Safety, Guangzhou 510000, China*

^c^*National Earthquake Emergency Rescue Training Base, Beijing 100059, China*

*e Work Safety Key Lab on Prevention and Control of Gas and Roof Disasters for Southern Coal Mines (Hunan University of Science and Technology), Xiangtan 411201, China*

**Definition**

**Risk factors**

It is the condition that guides the occurrence of a risk accident or increases the loss when a risk accident occurs.

**Safety guarantee**

It refers to the adoption of various measures to ensure the safety of people, property and information.

**Structural modeling method**

It is a functional modeling tool, which is mainly used to describe and analyze the structure and relationship between components of a system or organization. The focus of the structural model is on the objects or components that make up the system and their connections, rather than on the behavior or functions of these objects.

**Management defects**

In safety science, management defects refer to the shortcomings existing in safety management, which may lead to potential accidents or accidents. Management defects may involve various aspects, such as imperfect safety management system, unclear safety management responsibility, inadequate safety monitoring measures, insufficient safety training, etc. These defects may lead to the failure of effective control of dangerous factors, or the inability to respond in a timely manner in case of accidents, resulting in casualties and property losses.

**Description of the factors**

(1) Qualifications of relevant personnel (M_1_)

Personnel qualification primarily refers to the physical fitness of trainees. It should meet specific national/industry standards. Instructors are required to hold industry-recognized training qualification certificates, and trainees undergoing physical and practical training must be at an intermediate or advanced level. Two aspects are considered: physical fitness of personnel (A_1_) and safety education (A_2_)

(2) Equipment (M_2_)

Equipment mainly refers to the normal functioning of equipment used during skill and practical training. It should meet training needs and comply with relevant national/industry standards. Additionally, during routine checks before equipment deployment, it should start normally, be in good condition, and exhibit no faults. Two aspects are considered: equipment function (A_3_) and Equipment inspection (A_4_).

(3) Training facilities stability (M_3_)

The stability of the training facilities, especially in building collapses scenarios, should be ensured to prevent major malfunctions during their lifespan. Serious tilting or large-scale collapses causing damage could hinder training progress and pose a threat to the safety of trainees. Two aspects are considered: scientific design and construction (A_5_) and facility inspection and maintenance (A_6_).

(5) Personnel and organizational structure (M_4_)

Personnel and organizational structure involve balanced ratios of personnel in different positions, a complete organizational management structure, and the establishment of a comprehensive command system. This ensures that management systems are effectively implemented and facilitates smooth communication among personnel. Two aspects are considered: personnel ratio (A_7_) and organization system (A_8_).

A reasonable personnel ratio (A_7_) can improve training efficiency under the premise of fully guaranteeing safety. The personnel here mainly refer to instructors, trainees, doctors and security officers. As for organization system (A_8_), it must be ensured that the information communication between the training personnel is smooth. The instruction can be conveyed quickly and timely, and the trainees can respond quickly according to the instruction and establish a complete chain of command system.

(5) Health care system (M_5_)

The medical support system mainly refers to the completeness of Emergency supplies and plans (A_9_). The sufficiency of emergency medical supplies and equipment (such as Automated External Defibrillators - AEDs and on-site ambulances), clear delineation of responsibilities in emergency plans, and the clarity of emergency procedures directly impact whether casualties receive timely treatment.

(6) Personnel fault (M_6_)

During the training, trainees may make errors due to nervousness, lack of proficiency, or habitual actions. Particularly, the possibility of errors is highest for trainees conducting practical training for the first time. Two aspects are considered: operating standards and proficiency (A_10_), safety supervisor competence (A_11_).

(7) Facility risk (M_7_)

Facility risk primarily refers to the threats to personnel safety caused by fixed hazards within training facilities or factors such as the lack of detection and monitoring equipment. Two aspects are considered: potential fixed hazards in the facility (A_12_), Detection and monitoring coverage (A_13_).

There are some Potential fixed hazards in the facility (A_12_), such as sloping floors, exposed steel bars, uneven roads, cracks or sharp corners of cement and stone. They are used to improve the trainee’s anti-dizziness ability, but they are particularly prone to personal injury accidents such as slipping and falling in this environment. Detection and monitoring coverage (A_13_) mainly refers to the field of view that managers can effectively monitor through monitoring equipment. It is generally determined by the number of fixed cameras, installation location and monitoring duration, and the number of mobile detection devices.

(8) Equipment reliability (M_8_)

Equipment reliability indicates that the training equipment and personal protective equipment used during training possess reliable protective capabilities, operate normally, have high reliability, and low failure rates. Three aspects are considered: equipment protection effectiveness (A_14_), communication anti-interference ability (A_15_), intact personal protective equipment (A_16_).

(9) Operating environment risk (M_9_)

Operating environment risk indicates the threat to trainees from adverse factors in the surrounding environment during physical and practical training. Two aspects are considered: chronic occupational hazards (A_17_) and weather influences (A_18_).

(10) Implementation of a safety management system (M_10_)

Implementation of a safety management system indicates that trainees strictly follow established rules and regulations. Each individual should assume their respective responsibilities. With a well-established safety management system, the degree of implementation plays a crucial role in the management and personnel protection at the training site. Two aspects are considered: on-site management and control (A_19_), initiation efficiency of emergency plans (A_20_).

On-site management and control (A_19_) refers to restriction in the training area. Delimit the training scope, pull up the warning line, and control the entry and exit of relevant personnel and vehicles, which can improve the training efficiency and protect the safety of observers. Efficiency of emergency plans (A_20_). means that when the accident suddenly occurs, training safety responsible person, training safety manager, training instructor, security officer, medical support group, logistics support group, communication group, etc. will arrive at the designated location according to the training emergency plan.

(11) Maintenance, recovery, and recording (M_11_)

Maintenance, recovery, and recording indicate that, after training, necessary checks and maintenance are conducted on training facilities, equipment, and gear used during the training. Faulty equipment or gear is identified and sent for repairs before being returned to storage. Performance evaluations and summaries for trainees are also recorded. Two aspects are considered: equipment warehousing maintenance and records (A_21_), recovery and recording after training (A_22_).

(12) Mental health (M_12_)

Mental health indicates that trainees, after a certain duration of training (usually a day), may experience work-related stress and mental burdens due to the intensity of training or personal issues. It is detrimental to their psychological health. This can be reflected through rotation times and mental burden (A_23_).

**Table S1.** Influencing degree, influenced degree, centrality degree, and cause degree of each factor.

| **Factors** | **Influencing Degree (*D_i_*)** | **Ranking** | **Influenced Degree (*E_i_*)** | **Ranking** | **Centrality (*F_i_*)** | **Ranking** | **Causality (Mi)** | **Ranking** |
| --- | --- | --- | --- | --- | --- | --- | --- | --- |
| A_1_ | 0.0281 | 18 | 0.0617 | 8 | 0.0898 | 14 | -0.0336 | 19 |
| A_2_ | 0.1371 | 3 | 0.0002 | 23 | 0.1373 | 8 | 0.1370 | 2 |
| A_3_ | 0.0449 | 12 | 0.0741 | 6 | 0.1190 | 11 | -0.0293 | 15 |
| A_4_ | 0.1887 | 1 | 0.0263 | 17 | 0.2150 | 4 | 0.1625 | 1 |
| A_5_ | 0.0398 | 14 | 0.0368 | 16 | 0.0766 | 18 | 0.0030 | 12 |
| A_6_ | 0.0430 | 13 | 0.0454 | 12 | 0.0884 | 15 | -0.0024 | 13 |
| A_7_ | 0.0545 | 11 | 0.0369 | 15 | 0.0914 | 13 | 0.0175 | 8 |
| A_8_ | 0.0988 | 4 | 0.0237 | 18 | 0.1225 | 9 | 0.0752 | 4 |
| A_9_ | 0.0805 | 6 | 0.0399 | 14 | 0.1204 | 10 | 0.0406 | 6 |
| A_10_ | 0.1617 | 2 | 0.0548 | 10 | 0.2164 | 3 | 0.1069 | 3 |
| A_11_ | 0.0843 | 5 | 0.0735 | 7 | 0.1577 | 7 | 0.0108 | 11 |
| A_12_ | 0.0189 | 21 | 0.0421 | 13 | 0.0610 | 19 | -0.0233 | 14 |
| A_13_ | 0.0306 | 17 | 0.0183 | 20 | 0.0490 | 22 | 0.0123 | 10 |
| A_14_ | 0.0646 | 9 | 0.1124 | 3 | 0.1770 | 5 | -0.0478 | 20 |
| A_15_ | 0.0269 | 19 | 0.0566 | 9 | 0.0835 | 16 | -0.0297 | 16 |
| A_16_ | 0.0713 | 7 | 0.1045 | 4 | 0.1757 | 6 | -0.0332 | 18 |
| A_17_ | 0.0195 | 20 | 0.0792 | 5 | 0.0987 | 12 | -0.0596 | 21 |
| A_18_ | 0.0613 | 10 | 0.1676 | 2 | 0.2288 | 2 | -0.1063 | 22 |
| A_19_ | 0.0142 | 22 | 0.0464 | 11 | 0.0606 | 20 | -0.0321 | 17 |
| A_20_ | 0.0000 | 23 | 0.2636 | 1 | 0.2636 | 1 | -0.2636 | 23 |
| A_21_ | 0.0705 | 8 | 0.0090 | 22 | 0.0795 | 17 | 0.0616 | 5 |
| A_22_ | 0.0339 | 16 | 0.0144 | 21 | 0.0483 | 23 | 0.0196 | 7 |
| A_23_ | 0.0341 | 15 | 0.0201 | 19 | 0.0542 | 21 | 0.0140 | 9 |

**Table S2.** Reachable set and the antecedent set of each factor.

| **Factors** | **Reachable Set *R_i_*** | **Antecedent Set *W_i_*,** | **$R_{i}\cap W_{i}$** | **$R_{i}=R_{i}\cap W_{i}$** |
| --- | --- | --- | --- | --- |
| A_1_ | A_1_、A_18_ | A_1_、A_17_、A_18_、A_23_ | A_1_、A_18_ | A_1_、A_18_ |
| A_2_ | A_2_、A_6_、A_10_、A_11_、A_20_ | A_2_ | A_2_ |  |
| A_3_ | A_3_、A_14_、A_15_ | A_3_、A_4_、A_18_、A_21_ | A_3_ |  |
| A_4_ | A_3_、A_4_、A_9_、A_14_、A_15_、A_16_、A_18_ | A_4_、A_10_ | A_4_ |  |
| A_5_ | A_5_、A_12_、A_18_ | A_5_、A_6_、A_12_ | A_5_、A_12_ |  |
| A_6_ | A_5_、A_6_、A_12_ | A_2_、A_6_、A_10_ | A_6_ |  |
| A_7_ | A_7_、A_8_、A_20_ | A_7_、A_8_、A_11_ | A_7_、A_8_ |  |
| A_8_ | A_7_、A_8_、A_19_、A_20_ | A_7_、A_8_ | A_7_、A_8_ |  |
| A_9_ | A_9_、A_18_、A_20_ | A_4_、A_9_ | A_9_ |  |
| A_10_ | A_4_、A_6_、A_10_、A_11_、A_14_、A_16_、A_20_ | A_2_、A_10_ | A_10_ |  |
| A_11_ | A_7_、A_11_、A_16_、A_20_ | A_2_、A_10_、A_11_ | A_11_ |  |
| A_12_ | A_5_、A_12_ | A_5_、A_6_、A_12_ | A_5_、A_12_ | A_5_、A_12_ |
| A_13_ | A_13_、A_19_、A_20_ | A_13_ | A_13_ |  |
| A_14_ | A_14_、A_17_、A_18_ | A_3_、A_4_、A_10_、A_14_、A_21_ | A_14_ |  |
| A_15_ | A_15_、A_20_ | A_3_、A_4_、A_15_、A_21_ | A_15_ |  |
| A_16_ | A_16_、A_17_、A_18_、A_20_ | A_4_、A_10_、A_11_、A_16_ | A_16_ |  |
| A_17_ | A_1_、A_17_ | A_14_、A_16_、A_17_ | A_17_ |  |
| A_18_ | A_1_、A_3_、A_18_ | A_1_、A_4_、A_5_、A_9_、A_14_、A_16_、A_18_、A_22_、A_23_ | A_1_、A_18_ |  |
| A_19_ | A_19_、A_20_ | A_8_、A_13_、A_19_ | A_19_ |  |
| A_20_ | A_20_ | A_2_、A_7_、A_8_、A_9_、A_10_、A_11_、A_13_、A_15_、A_16_、A_19_、A_20_ | A_20_ | A_20_ |
| A_21_ | A_3_、A_14_、A_15_、A_21_ | A_21_ | A_21_ |  |
| A_22_ | A_18_、A_22_ | A_22_ | A_22_ |  |
| A_23_ | A_1_、A_18_、A_23_ | A_23_ | A_23_ |  |
